# Diagnostische Herausforderungen des Korsakow-Syndroms: Ein Fallbericht

**DOI:** 10.1007/s40211-025-00566-y

**Published:** 2025-12-16

**Authors:** Paula Hoppstock, Lea Sommer, Mathias Jachs, Clemens Dejaco, Gregor Kasprian, Radheshyam Stepponat, Armin Trojer, Sabine Weber, Stephan Listabarth, Daniel König

**Affiliations:** 1 Klinische Abteilung für Sozialpsychiatrie, Universitätsklinik für Psychiatrie und Psychotherapie, Medizinische Universität Wien, Währinger Gürtel 18–20, 1090 Wien, Österreich; 2 Comprehensive Center for Clinical Neurosciences and Mental Health (C3NMH), Medizinische Universität Wien, Wien, Österreich; 3 Klinische Abteilung für Gastroenterologie und Hepatologie, Univ.-Klinik für Innere Medizin III, Medizinische Universität Wien, Wien, Österreich; 4 Klinische Abteilung für Neuroradiologie und Muskuloskeletale Radiologie, Univ.-Klinik für Radiologie und Nuklearmedizin, Medizinische Universität Wien, Wien, Österreich

**Keywords:** Thiaminmangel, Alkoholkonsumstörung, Wernicke-Korsakow-Syndrom, Atrophie der corpora mamillaria, osmotisches Demyelinisierungssyndrom, Thiamine deficiency, Alcohol use disorder, Wernicke-Korsakoff syndrome, Mamillary body atrophy, Osmotic demyelination syndrome

## Abstract

Das Korsakow-Syndrom (KS) stellt in Diagnose und Versorgung eine interdisziplinäre Herausforderung dar, wobei primär das häufige Fehlen eindeutiger klinischer Symptome eine frühzeitige Diagnose erschweren kann. Dieser Fallbericht beschreibt einen 59-jährigen Patienten, der sich aufgrund seiner klinischen Symptomatik zunächst in internistischer Behandlung befand, da kardiovaskuläre und metabolische Komorbiditäten eine komplexe Diagnostik notwendig machten. Die primäre Verdachtsdiagnose eines, in der klinischen Symptomatik oft ähnlich dem KS imponierenden, osmotischen Demyelinisierungssyndroms (früher: pontine Myelinolyse) konnte erst durch den MRT-Nachweis einer Atrophie der Corpora mamillaria ausgeschlossen und schließlich die Diagnose eines KS gesichert werden. Unter hochdosierter Thiamin-Supplementation, neurorehabilitativen Maßnahmen und mit Unterstützung durch ein stabiles soziales Umfeld zeigte sich im Verlauf eine Verbesserung der kognitiven Leistungsfähigkeit. Der Fall hebt die Notwendigkeit einer differenzialdiagnostischen Abklärung des KS sowie einer interdisziplinären Sensibilisierung über neurologische und psychiatrische Fachdisziplinen hinaus, hervor.

## Abstract (en)

Korsakoff syndrome (KS) presents an interdisciplinary challenge in diagnosis and care. The frequent lack of distinct clinical symptoms represents a diagnostic challenge and can complicate early diagnosis. This case report describes a 59-year-old patient who initially presented to internal medicine due to symptoms that appeared to be associated with pre-known cardiovascular and metabolic comorbidities. The initial suspected diagnosis of osmotic demyelination syndrome (formerly known as pontine myelinolysis), which can induce a clinical state very similar to KS, could only be ruled out by MRI evidence of atrophy of the mammillary bodies, confirming the diagnosis of KS. High-dose thiamine supplementation, neurorehabilitative measures, and support by a stable social environment, led to an improvement in cognitive performance. This case highlights the need for differential diagnostic efforts regarding KS and interdisciplinary awareness beyond neurological and psychiatric disciplines.

## Einleitung

Alkohol ist, insbesondere in Europa, ein bedeutender Risikofaktor für Morbidität und Mortalität [1]. Laut WHO leiden weltweit schätzungsweise 7 % der Bevölkerung ab 15. Lebensjahr – also etwa 400 Mio. Menschen, an einer Alkoholkonsumstörung (entsprechend der Einteilung nach DSM‑5 [englisch: „*alcohol use disorder“*], die sowohl schädlichen Gebrauch als auch Abhängigkeit umfasst und damit über die Terminologie der ICD-10 hinausgeht). Davon erfüllen über 200 Mio. Menschen (3,7 % der Weltbevölkerung ab dem 15. Lebensjahr) die Kriterien einer Alkoholabhängigkeit [2]. Alkohol hat erhebliche Auswirkungen auf den Körper und beeinträchtigt sowohl die Resorption als auch den Metabolismus von Thiamin (Vitamin B1) [3, 4]. Thiamin ist ein wasserlösliches Vitamin, welches als essenzielles Coenzym eine wesentliche Rolle im Kohlenhydratstoffwechsel, insbesondere im Gehirns, spielt [4]. Ein Mangel an Thiamin kann potenziell tödliche Folgen haben und kann ursächlich für die Wernicke Enzephalopathie (WE) – ein Zustand mit potenziell tödlichem Verlauf – und das Korsakow-Syndrom (KS) sein. Beide Erkrankungsbilder sind in Europa primär mit einer Alkoholkonsumstörung assoziiert [4, 5].

Die Prävalenz der WE in der weltweiten Allgemeinbevölkerung wird auf 0,4 bis 2,8 % geschätzt [5], wobei die klassische Symptomtrias aus Verwirrtheit, Ataxie und Ophthalmoplegie [6] nur bei 16–33 % der betroffenen Patient:innen vorliegt [5]. Bereits das Auftreten eines dieser Symptome, insbesondere bei gleichzeitigem Vorliegen von bekannten Risikofaktoren (wie Alkoholkonsumstörungen), sollte den Verdacht auf eine WE begründen und eine sofortige Thiamin-Gabe sowie weitere Diagnostik veranlassen [3, 4, 7]. Eine adäquate Thiaminsubstitution stellt die entscheidende therapeutische Maßnahme bei der Behandlung der WE und kann, bei rechtzeitiger Durchführung, zu einer vollständigen Remission führen und die Progression zum KS verhindern [8]. Obwohl die Substitution von Thiamin in den Leitlinien vorgesehen ist, wird sie in der klinischen Praxis immer noch nicht durchgehend durchgeführt [9]. Dies liegt zum Teil daran, dass die Evidenzlage zu Dauer und Dosierung noch limitiert ist und daher klare Empfehlungen zu Dosierung, Frequenz, Verabreichungsweg oder Dauer der Thiaminbehandlung fehlen [10].

Bei Ausbleiben der Thiaminsubstitution kann sich ein KS entwickeln. Die Diagnose dieser schwerwiegenden neuropsychiatrischen Störung wird in Ermangelung spezifischer und unmittelbar verfügbarer Labor- und Bildgebungsmarker primär klinisch gestellt [11–13]. Das KS ist durch eine schwerwiegende antero- und/oder retrograden Amnesie bei erhaltenem Kurzzeitgedächtnis [14, 15] gekennzeichnet und häufig von Konfabulationen begleitet, bei denen Betroffene unbewusst Gedächtnislücken mit fiktiven Inhalten füllen [11, 14]. Zusätzlich können eine zeitliche und örtliche Desorientierung [16, 17], Verwirrung [12, 13, 17] sowie Defizite in exekutiven Funktionen auftreten [16, 18].

Die diagnostische Abklärung der WE und des KS gestalten sich aufgrund des heterogenen klinischen Erscheinungsbildes oft schwierig [11, 13, 19] – vor allem, da initial eine gewisse Ähnlichkeit in der Symptomatik zu einem Delir [12] oder einem osmotischem Demyelinisierungssyndrom (früher: pontine Myelinolyse) bestehen [20] und diese Syndrome ebenso im Kontext von Alkoholkonsumstörung auftreten können [12, 20].

Das osmotische Demyeliniserungssyndrom (ODS) kann infolge einer zu raschen Korrektur einer chronischen Hyponatriämie auftreten. Die Magnetresonanztomographie gilt als der Goldstandard in der Diagnostik des ODS [21]. Basierend auf der S1-Leitlinie sollte bei der Korrektur einer Hyponatriämie, besonders bei Risikopatient:innen mit Alkoholkonsumstörung oder Mangelernährung, eine maximale Anhebung des Serum-Natriumspiegels von 6– 12 mmol/l innerhalb von 24 h nicht überschritten werden, um das Risiko eines osmotischen Demyelinisierungssyndroms zu minimieren [22, 23]. Chronischer Alkoholkonsum und Alkoholentzug gelten als Komorbiditäten, die das Risiko für ein osmotisches Demyelinisierungssyndrom erhöhen [24]. Das klinische Bild des ODS ist variabel und umfasst Symptome einer Enzephalopathie, darunter Bewusstseinsstörungen, Delirium sowie Antriebs‑, Gedächtnis- und Konzentrationsstörungen, wobei auch asymptomatische oder oligosymptomatische Verläufe möglich sind [24]. Wenngleich keine exakten epidemiologischen Daten zum ODS existieren, beschreiben MRT-basierte Studien eine Inzidenz von 0,3 % bis 1,1 %, wobei unter Hochrisikopatient:innen Inzidenzen von 29 % beschrieben wurden [24].

Dieser Fall unterstreicht die Bedeutung der differenzialdiagnostischen Abklärung von alkoholassoziierten Folgeerkrankungen bei Patient:innen mit Alkoholkonsumstörung – insbesondere bei zugleich bestehender Mangelernährung sowie neuropsychiatrischer Symptomatik [25, 26]. Weiters hebt der vorliegende Fallbericht den Stellenwert frühzeitiger Interventionen und neurorehabilitativer Maßnahmen hervor, um lebensbedrohliche Komplikationen zu reduzieren und Folgeschäden so gering wie möglich zu halten [3, 12].

## Kasuistik

### Internistischer Verlauf

Ein 59-jähriger Patient wurde aufgrund von progredienter Schwäche und Schwindel nach einer einwöchigen Episode von Diarrhö und Emesis akut mit der Rettung an der Univ.-Klinik für Notfallmedizin der Medizinischen Universität Wien vorgestellt. Die Exfrau des Patienten hatte den Patienten in deutlich reduziertem Allgemeinzustand in seiner Wohnung vorgefunden und einen Schlaganfall vermutet. Zuletzt hatte sie ihn eine Woche zuvor in orientiertem Zustand und, abgesehen von Diarrhö, klinisch beschwerdefrei gesehen. In den Folgetagen habe er, während der regelmäßig stattgefundenen telefonischen Kontakte, zunehmend verwirrt imponiert. Bei dem Patienten war eine Alkoholabhängigkeit vorbekannt.

In der Univ.-Klinik für Notfallmedizin präsentierte sich der Patient kachektisch, ikterisch und zeitlich, örtlich sowie situativ desorientiert. Neben dem reduzierten Allgemein- und Ernährungszustand zeigte sich eine metabolische Entgleisung im Sinne einer Hyponatriämie (125 mmol/l; Referenzbereich [Ref.]: 136–145 mmol/L), einer eingeschränkten Nierenfunktion (GFR 22 ml/min; Ref.: > 55 ml/min, BUN 80 mg/dl; Ref.: 6–20 mg/dl) sowie einer Serumlaktatauslenkung (3 mmol/l; Ref.: 0,5–2,2 mmol/l). Trotz klinischem Ikterus zeigte sich laborchemisch kein erhöhtes Bilirubin (0,72 mg/dl; Ref.: 0–1,2 mg/dl), sondern lediglich eine γ‑GT-Erhöhung (96 U/l; Ref.: < 40 U/l) und eine erniedrigte Cholinesterase (2,55 kU/l; Ref.: 5,32–12,92 kU/l). Zudem wurden bereits auf der Univ.-Klinik für Notfallmedizin laborchemisch erhöhte Pankreasenzyme (Alpha-Amylase 368 U/L; Ref.: 28–100 U/L; Lipase 583 U/L; Ref.: 13–60 U/L) festgestellt – vereinbar mit einer alkoholinduzierten Pankreatitis sowie einem akuten (prärenalen) Nierenversagen.

Die beobachtete Desorientierung wurde im Kontext der metabolischen Entgleisung als Zeichen eines Delirs interpretiert. Ein präsentierter Halte- und Intentionstremor wurde einem Alkoholentzugssyndrom zugeschrieben. Die klinische neurologische Abklärung ergab zunächst keine weiteren Auffälligkeiten. Zur weiteren Betreuung wurde der Patient an der klinischen Abteilung für Gastroenterologie und Hepatologie der Univ.-Klinik für Innere Medizin III der Medizinischen Universität Wien (MUW) am Allgemeinen Krankenhaus der Stadt Wien (AKH Wien) aufgenommen. Dort zeigte sich ein Harnwegsinfekt mit Leukozyturie, der in diesem Zusammenhang als zusätzlicher Risikofaktor für eine delirante Symptomatik gesehen und entsprechend behandelt wurde. Der Behandlungsverlauf des Patienten umfasste neben der intravenösen Flüssigkeitsgabe auch eine antibiotische Therapie mit Cefotaxim. In weiterer Folge konnte eine internistische Stabilisierung erreicht werden. Aufgrund der bekannten Alkoholabhängigkeit erhielt der Patient, entsprechend den Leitlinien, hochdosiert Thiamin (Thiaminsubstitution mit 100 mg i.v. in 100 ml NaCl 0,9 %, dreimal täglich über 5 Tage) (Tab. 1). Zusätzlich wurde im Rahmen des Alkoholentzugs zur Kupierung der Beschwerden Oxazepam etabliert. Anhaltende kognitive Verlangsamung, eine auffällige Dysarthrie sowie wiederholte sturzbedingte Verletzungen führten zu der Etablierung von Melperon zur symptomatischen Therapie bei Alkoholentzugsdelir (in Österreich für die Behandlung des Alkoholentzugsdelirs zugelassen). Im Verlauf erfolgte die Verlegung des Patienten zur Fortsetzung des medikamentös gestützten Alkoholentzug auf die klinische Abteilung für Sozialpsychiatrie der Universitätsklinik für Psychiatrie und Psychotherapie.

**Tab. 1:** Empfehlungen der Thiaminsubstitution

Thiaminsubstitution	Empfohlenes Substitutionsshema	Anmerkung
*Akuttherapie (i.v.)*	100 mg Thiamin i.v. in 100 ml NaCl 0,9 %, dreimal täglich über 5 Tage	Frühzeitig und hochdosiert
*Akuttherapie (p.o.)*	100 mg Thiamin p.o., dreimal täglich über 7 Tage	Bei Kontraindikation gegen i.v.-Gabe oder auf Patientenwunsch
*Erhaltungstherapie*	100 mg Thiamin p.o. täglich	Dauerhafte orale Substitution empfohlen

Modifiziert nach Listabarth et al. [8]

### Psychiatrischer Verlauf

Bei Übernahme zeigte der Patient bei Erhebung von Anamnese und Status deutliche Gedächtnislücken – sowohl hinsichtlich der Umstände seiner Einweisung als auch bezüglich des Verlaufs des Krankenhausaufenthaltes. Er war zeitlich und örtlich kontinuierlich nur partiell orientiert (keine korrekte Datumsangabe bei gleichzeitig korrekter Monats- und Jahresangabe; korrekte allgemeine Ortsangabe: Krankenhaus, Name des Krankenhauses nicht korrekt angegeben). Psychopathologisch ergaben sich bis auf diese kognitiven Defizite keine weiteren Auffälligkeiten. Neurologisch hingegen bestanden bei Übernahme eine Dysarthrie sowie eine Gangataxie als Hinweis auf eine zerebelläre Dysfunktion. Zudem zeigte sich beidseits eine leichte Bradydiadochokinese sowie ein Endstellnystagmus bei der Prüfung der Okulomotorik. Weitere neurologische Auffälligkeiten konnten nicht festgestellt werden. Zwar präsentierte sich der Patient mit Defiziten in der Gedächtniskonsolidierung, diese waren jedoch nur partiell vorhanden.

Die Alkoholanamnese ergab einen langjährigen täglichen Konsum von durchschnittlich 10–15 Bier à 500 ml entsprechend einer Menge von mindestens 200–300 g reinen Alkohols täglich, sowie zusätzlich hochprozentigen Alkohol in unbestimmter Menge, wobei der Patient angab mit dem Konsum bereits frühmorgendlich zu beginnen. Wiederholte stationäre Entzugsbehandlungen hatten in der Vergangenheit stattgefunden, von denen eine zu einer sechsmonatigen Abstinenz geführt hatte, während zwei aufgrund eines frühzeitigen erneuten Konsums abgebrochen wurden. Lebenszeitlich wurden epileptische Geschehen sowie entzugsdelirante Zustandsbilder negiert. Der Patient lebte zu dem Zeitpunkt der Aufnahme in das Spital in einem stabilen sozialen Umfeld, das ihn sowohl finanziell als auch in alltäglichen Angelegenheiten unterstützte.

Aufgrund der Initialsymptomatik mit Mangelernährung, Hypovolämie mit Hyponatriämie bei Diarrhö, Dysarthrie, Dysphagie, Orientierungsstörung sowie der Elektrolytsubstitution und der Cefotaximgabe (cave: Natriumreiches Präparat [27]) an der Univ.-Klinik für Notfallmedizin der Medizinischen Universität Wien, wurde in der differenzialdiagnostischen Überlegung zwischen dem osmotischen Demyelinisierungssyndrom (ODS; früher: pontine Myelinolyse) oder ein KS erwogen. Aufgrund der relativ milden kognitiven Beeinträchtigung ging man allerdings von der Diagnose eines alkoholbedingten ODS ausgegangen (Tab. 2 und 3).

**Abb. 1:**
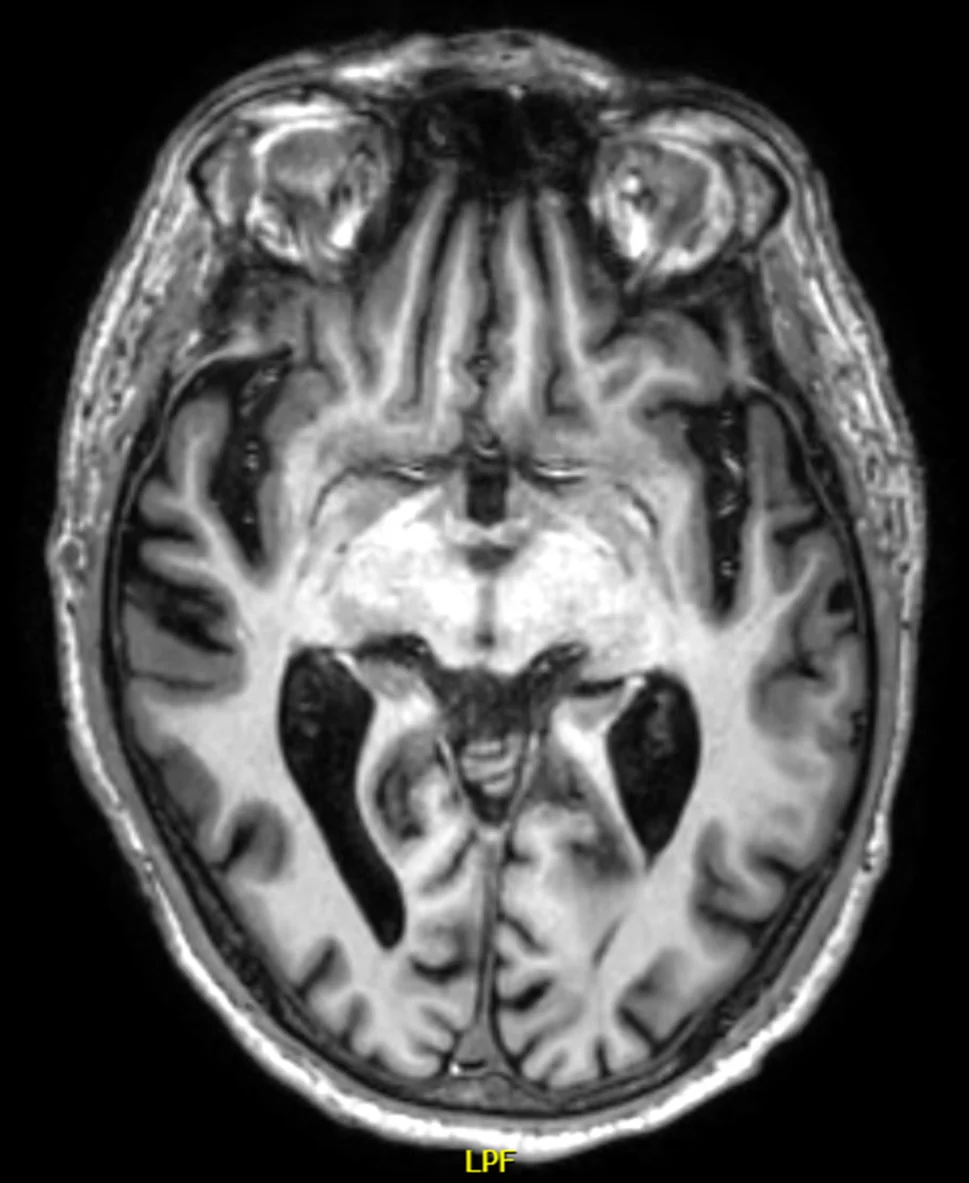
T1-gewichtete axiale zerebrale Magnetresonanztomographie-Aufnahme zeigt deutliche Atrophie der Copora mamillaria

Die zerebrale Magnetresonanztomographie (cMRT) zeigte eine hochgradige Atrophie der Corpora mamillaria. Dieser Befund sprach in Zusammenschau mit der klinischen Präsentation für ein KS, wenngleich die relativ milde neuropsychiatrische Symptomatik dies zunächst nicht als primäre Verdachtsdiagnose erscheinen ließ. Weiters fanden sich im cMRT alkoholassoziierte strukturelle Hirnveränderungen mit supra- und infratentorieller Atrophie, zerebellärer Beteiligung sowie mikroangiopathischen Läsionen im Sinne einer Leukenzephalopathie Grad II nach Fazekas und lakunären Infarkten (Tab. 4, Abb. 1).

**Tab. 2:** Vergleich der Symptome des Korsakow-Syndrom und osmotisches Demyelinisierungssyndrom

Symptomkategorie	Korsakow-Syndrom (einschließlich möglicher Residualsymptome nach Wernicke-Enzephalopathie)	Symptomkategorie	Osmotisches Demyelinisierungssyndrom
**Gedächtnis**		**Gedächtnis**	Unspezifische Gedächtnisstörung, in der Literatur nicht näher beschrieben [21]
Episodisches Gedächtnis	(Insbesondere) anterograde Amnesie [14], retrograde Amnesie [14], Konfabulationen [6, 13]		
Kontext‑/Quellengedächtnis	Zeitlich-räumliche Einbettung stark gestört [14]		
Semantisches Gedächtnis (Altgedächtnis)	Alte Fakten meist erhalten, aber Neulernen von Konzepten beeinträchtigt [14]		
Autobiografisches sem. Gedächtnis (Altgedächtnis)	z. B. Adresse in der Kindheit meist erhalten, spätere biografische Fakten teils verloren [14]		
**Bewusstseinsstörung/Enzephalopathie**	Zeitliche und örtliche Desorientierung, Verwirrung [11, 14]	**Bewusstseinsstörung/Enzephalopathie**	Vigilanzstörung, Delir, Koma [21]
**Exekutive/frontale Dysfunktion**	Beeinträchtigung von Planungs‑, Organisations‑, Problemlösungs- und Entscheidungsfähigkeiten [7, 11]	**Exekutive/frontale Dysfunktion**	Problemlösen, Handlungsplanung, Antrieb, Impulskontrolle, Konzentrationsstörungen [21]
**Affekt und Verhalten**	Apathie, emotionale Instabilität, Frontalhirn-Disinhibition, depressive Symptome [14]	**Affekt und Verhalten**	Apathie, Lethargie, Depressive Symptome, Manische Symptome, Katatonie, Mutismus, Antriebsstörung, Impulskontrollstörung, emotionale Regulation [21]
**Bewegungsstörung**	*Residualsymptom nach Wernicke-Enzephalopathie möglich: Gangataxie* [11]	**Bewegungsstörung (extrapyramidal-motorische)**	Akinetisch-rigide Symptome, Tremor, Dystonie, Chorea, Ataxie, Myoklonus, Opsoklonus, Gangstörungen [21]
		**Motorik/Pyramidenbahn**	Paresen, Areflexie [21]
**Hirnstammsyndrom**	*Residualsymptom nach Wernicke-Enzephalopathie möglich: Augenmotilitätsstörung, Nystagmus* [4]	**Hirnstammsyndrom**	Locked-in-Syndrom, Dysarthrie, Dysphagie, Augenmotilitätsstörung [21]

**Tab. 3:** Vergleich Korsakow-Syndrom und osmotisches Demyelinisierungssyndrom: Vorstufen, Therapieansatz und Therapieempfehlung

Krankheitsbild	Vorstufe	Therapeutischer Ansatz	Präventive Therapieempfehlung	Anmerkung
*Korsakow-Syndrom*	Wernicke-Enzephalopathie	Prävention durch Thiaminsubstitution	Thiaminsubstitution: 100 mg Thiamin *i.v.* in 100 ml NaCl 0,9 %, dreimal täglich über 5 Tage, danach dauerhafte orale Substitution empfohlen (100 mg Thiamin *p.o.* täglich)	Frühzeitig und hochdosiert substituieren
*Osmotisches Demyelinisierungssyndrom*	Hyponatriämie	Prävention durch langsame Na-Korrektur	Natriumkorrekturrate: Erhöhung des Serum-Natriumspiegels maximal um 6 mmol/l innerhalb von 24 h	Langsam bis zu einem Serum-Natriumspiegel von 130 mmol/l ausgleichen

Modifiziert nach [8] für das Korsakow-Syndrom und nach [23] für das osmotische Demyelinisierungssyndrom

Während des stationären Aufenthalts ließ sich eine undulierend ausgeprägte (zumeist milde) Merkfähigkeitsstörung mit anterograder und retrograder Teilamnesie feststellen, erkennbar am intermittierenden Nichterkennen seiner Kinder und der Angabe einer nicht mehr aktuellen Wohnadresse. Zudem bestand weiterhin nur eine Teilorientierung zu Zeit und Ort, sodass die Thiamin-Substitution initial auf 600 mg/d gesteigert wurde. Eine ergänzende neuropsychologische Testung ergab bei durchschnittlicher kristalliner Intelligenz ein heterogenes Leistungsprofil mit Beeinträchtigungen des verbalen und nonverbalen Gedächtnisses, der Verarbeitungsgeschwindigkeit sowie der mentalen Flexibilität, während Exekutivfunktionen und verbale Flüssigkeit unauffällig waren. Die Merkfähigkeitsstörungen führten zu Unterstützungsbedarf bei Körperpflege, An- und Auskleiden sowie der Nahrungsaufnahme. Im Verlauf zeigten sich durch neurorehabilitative Maßnahmen, einschließlich kognitivem Training, Physiotherapie sowie re-orientierenden Maßnahmen kognitive Verbesserungen in Alltagskompetenzen, eine moderate Regredienz der Dysarthrie sowie eine Verbesserung der Orientierung. Nach Organisation einer umfassenden ambulanten Unterstützung in pflegerischen, sozialen und alltagsbezogenen Bereichen konnte der Patient ins häusliche Umfeld entlassen werden. In einer erneuten ambulanten Begutachtung zwei Monate nach Entlassung konnte eine zunehmende Verbesserung des Zustandes beobachtet werden.

**Tab. 4:** Radiologische Zeichen – Korsakow-Syndrom

Stadium	Radiologische Zeichen im MRT
*Frühstadium *(Wernicke – Enzephalopathie)	*Signalalterationen *(Hyperintensitäten) im *limbischen System* (Thalami, Mammillarkörper, Fornices, periventrikuläres Grau)
*Chronisches Stadium *(Korsakow-Syndrom)	Deutlich stärkere *Atrophie der Mammillarkörper und Thalami*, kortikale Volumenminderung, Erweiterung der Sulci und Ventrikelerweiterung (Selten noch Signalveränderungen nachweisbar)

Modifiziert nach [28]

## Diskussion

Der vorliegende Fall veranschaulicht das KS als Komplikation einer chronischen Alkoholkonsumstörung. Obwohl bekannt ist, dass die Alkoholkonsumstörung die Entwicklung von WE und KS begünstigen kann, besteht das Risiko, dass das KS aufgrund unzureichender Sensibilisierung des medizinischen Personals verkannt und verzögert gestellt wird.

Post-mortem Analysen zeigen, dass WE bei etwa 1 % der Allgemeinbevölkerung und 12,5–35 % der Patient:innen mit bekannter Alkoholkonsumstörung auftritt [19]. Auch in diesem präsentierten Fall erschwerten multiple Komorbiditäten wie Hyponatriämie, Infektionen und (Prä‑)Delir die korrekte Diagnose, da sie überlappende Symptome wie Gedächtnis- und Orientierungsstörungen verursachten. Dies führte initial zum Verdacht eines osmotischen Demyeliniserungssyndroms alkoholtoxischer Genese.

Die Definition des KS ist weiterhin Gegenstand wissenschaftlicher Diskussion, insbesondere im Hinblick auf das Verhältnis von Gedächtnisstörungen zu anderen kognitiven Defiziten [14]. *Scalzo und Bowden* kritisieren die Fokussierung auf ein rein amnestisches Syndrom, da dies Fehldiagnosen bei Patient:innen mit variableren kognitiven Defiziten begünstigt [13]. Weiters weisen sie auf eine umfassendere diagnostische Perspektive hin, die Schweregrad und Chronizität berücksichtigt [13]. Auch im vorliegenden Fall war die Amnesie zunächst schwer fassbar, von einem fluktuierenden Verlauf und bestand lediglich als Teilamnesie, sodass sie auf der internistischen Station nicht unmittelbar auffiel.

Ausschlaggebend für die Diagnosesicherung war in dem geschilderten Fall die MRT Untersuchung, die im Akutsetting nicht immer verfügbar ist und eine limitierte Sensitivität aufweist [12]. Die Atrophie der Corpora mamillaria ist ein charakteristischer, aber nicht zwingend vorliegender neuroradiologischer Befund des KS [14]. In Übereinstimmung mit *Arts et al.* verdeutlicht dieser Fall die Bedeutung der neuropsychologischen Testung, um kognitive Defizite individuell zu erfassen und eine gezielte Therapie zu ermöglichen, da deren Ausprägung stark variieren kann [16].

Dieser Fall hebt die differenzialdiagnostischen Abklärung des KS bei Patient:innen mit Risikofaktoren und kognitiven Defiziten hervor und betont die Notwendigkeit einer frühzeitigen Thiamin-Supplementation [13]. Trotz oft persistierender kognitiver Defizite sind bei adäquater Therapie und Alkoholabstinenz Verbesserungen möglich [18], welche auch bei dem beschriebenen Patienten erreicht werden konnten. Die im Verlauf aufgetretene Infektion mit nachfolgendem Delir verdeutlicht die Vulnerabilität dieser Patient:innen und den Bedarf an engmaschiger Überwachung.

Die Diagnostik und Therapie des KS werden durch fehlende Standardisierung erschwert [18]. Die S3-Leitlinie der AWMF empfiehlt zwar 3 × 250–500 mg Thiamin i.v. pro Tag bei WE, die große Spannbreite verdeutlicht aber die bestehenden Herausforderungen [29]. Diese Limitationen unterstreichen den Bedarf an weiterer Forschung und standardisierten Behandlungsleitlinien für KS und WE. Die Caine-Kriterien haben die diagnostische Sensitivität für WE deutlich verbessert [30] und die Diagnoserate im Vergleich zur klassischen Trias um bis zu 62 % gesteigert [7]. Dies zeigt das Potenzial operationalisierter Kriterien für die Früherkennung und Behandlung von WE und KS.

Die intensive familiäre Unterstützung könnte im positiven Rehabilitationsverlauf dieses Patienten eine entscheidende Rolle gespielt haben. *Kopelman* beschreibt die Bedeutung regelmäßiger Familienbesuche sowie eines unterstützenden sozialen Umfelds für die Rehabilitation [31]. Strukturierte, alltagsnahe Rehabilitationsmaßnahmen, die durch soziale Unterstützung optimiert werden, sind besonders wertvoll [16]. Im vorliegenden Fall ermöglichte die familiäre Unterstützung eine kontinuierliche Förderung und Integration in ein strukturiertes Umfeld. Die Miteinbeziehung und Unterstützung des sozialen Umfeldes könnte im Sinne von Angehörigenarbeit wichtig sein. Dies könnte die beobachteten Verbesserungen der kognitiven Leistungsfähigkeit und Alltagskompetenz erklären und die Bedeutung sozialer Ressourcen im Rehabilitationsprozess unterstreichen [16, 31]. Die Förderung der Angehörigenarbeit könnte daher ein entscheidender Bestandteil der Rehabilitation sein, um soziale Ressourcen gezielt zu nutzen und langfristige Stabilität zu gewährleisten.

Um die Versorgung und Prognose von Patient:innen mit KS zu verbessern, ist eine Sensibilisierung aller medizinischen Fachrichtungen für dieses Syndrom essenziell, insbesondere da der Erstkontakt oft außerhalb der Neurologie oder Psychiatrie stattfindet [12, 16, 18].
